# Local Ras/actin signaling orients individual pseudopods in shallow gradients

**DOI:** 10.1073/pnas.2526852123

**Published:** 2026-03-06

**Authors:** Peter J. M. van Haastert

**Affiliations:** ^a^Department of Cell Biochemistry, University of Groningen, Groningen 9747 AG, The Netherlands

**Fig. 1.**
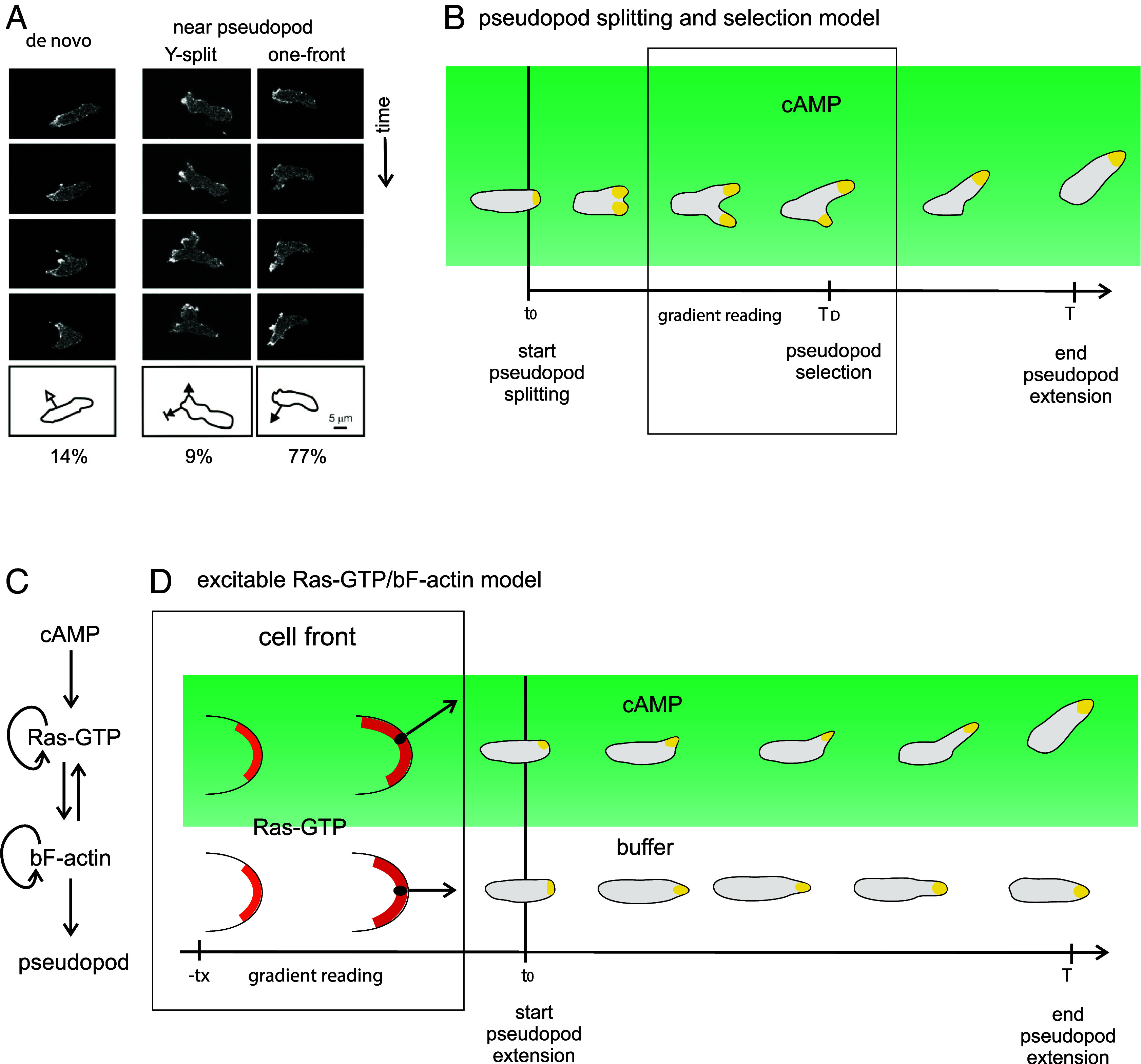
(*A*) Definition pseudopods. De novo pseudopods emerge from the side of the cell; most pseudopods start near existing pseudopods. “Y-split” emerges by bifurcation of the previous pseudopod; it has two extending fronts, of which one is retracted by competition (1). “One-front” pseudopods emerge at the side of the previous pseudopod (4); the cell has only one extending front. Image credit: Adapted from ref. 4. (*B*) Pseudopod splitting and selection model of Alonso et al. (3). Y-splitting pseudopods receive local gradient information (box), which stabilizes the pseudopod in the direction of the gradient. (*C* and *D*) Excitable Ras-GTP/bF-actin model of Van Haastert et al. (5). Box: a patch of excitable Ras-GTP/bF-actin grows in size and intensity during *t_x_* seconds (*t_x_* ≈ 3 s). In buffer, the patch grows symmetrically, but in a gradient, the patch grows more strongly at the side facing adenosine 3’,5’-monophosphate cAMP. The pseudopod starts at about the center of the patch perpendicular to the membrane curvature (black dot and arrow).

Eukaryotic cells navigate effectively in very shallow chemotactic gradients. The issue is how cells analyze such dim signals. Experiments suggest that cells extend pseudopods with a directional bias imposed by the local concentration of chemoattractant (1, 2). Alonso et al. (3) describe a simple mechanism for local signaling, based on earlier work by Andrew and Insall (1) on pseudopod splitting: The extending pseudopod splits into two pseudopods, of which one is retracted and the other becomes dominant. The local gradient stabilizes the pseudopod that is extended toward the gradient. Alonso et al. (3) provide an elegant model for pseudopod splitting/selection that is based on competition for a limiting pool of actin (Fig. 1*B*). However, experimental data indicate that pseudopod splitting is rare and actin is not limiting.

Stimulated by the work of Andrew and Insall (1), we investigated chemotaxis from the perspective of the extending pseudopod and reported in 2009 that true pseudopod splitting is rare (4): Only about 10% of the pseudopods are “Y-split” with two extending fronts; nearly all pseudopods are “one-front” with a single extending front (Fig. 1*A*). Since then, we collected data from about 12,000 pseudopods in four species (*Dictyostelium*, neutrophils, mesenchymal stem cells, and *B.d. chytrid*), in numerus mutants, and at many experimental conditions including chemotaxis and electrotaxis (6, 7). Indeed, Y-type splitting with two fronts is very rare (Table 1). Cells have a high probability to start a first pseudopod; the start of a second pseudopod is strongly inhibited (7); therefore, most cells (~76%) exhibit one pseudopod with one extending front (Table 1). Furthermore, several mutants have lost this inhibition and extend one to five pseudopods; size and extension time do not change with pseudopod number (7), indicating that actin is not a limiting factor for two splitting pseudopods (7).

**Table 1.** Pseudopods in buffer and a cAMP gradient

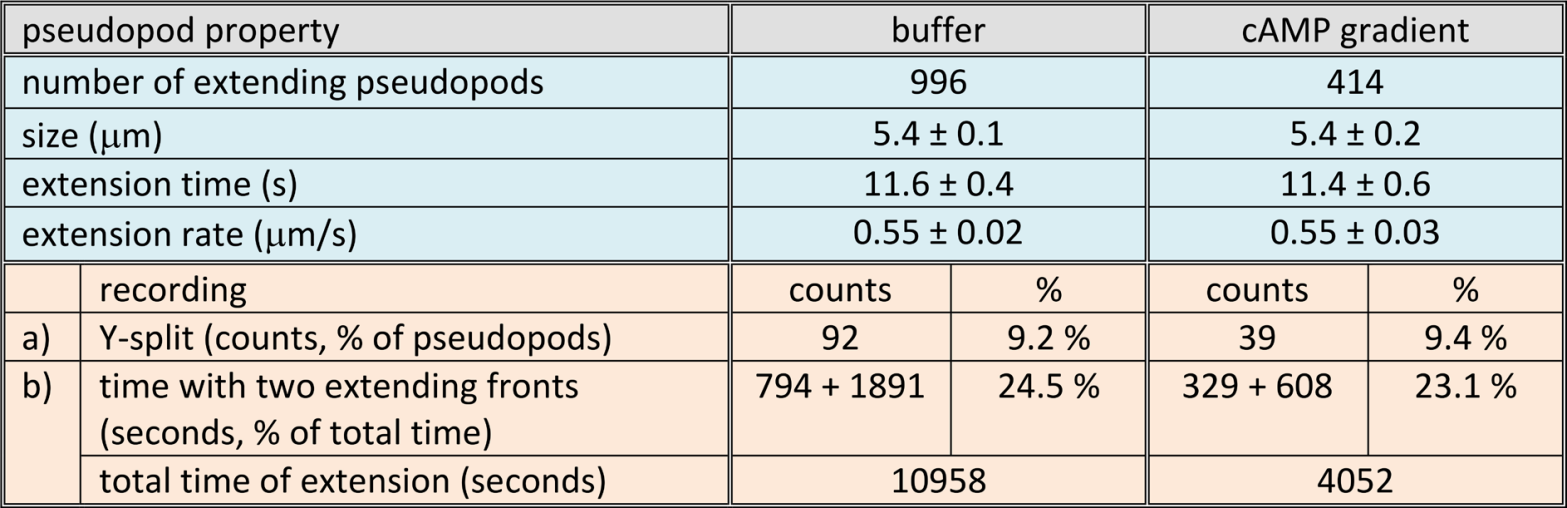

The data are derived from the experiments described in ref. 7. Movies were recorded at 1 frame per second. Pseudopods were identified and characterized by the (x,y,t) coordinates at the start and stop of their extension. Blue: Pseudopod properties. Data are means and 95% CI, derived from Table 1 in ref. 7. The differences between observed values in buffer and cAMP gradient are not significant at *P* > 0.1. Orange: Cells with multiple extending fronts. a), Y-split is a pseudopod with two extending fronts that emerge by bifurcation of a previous pseudopod (1); these pseudopods are the subject of the study of Alonso et al. (3). The data are counts of the number of pseudopods that comply to this definition; the percentage is this count divided by the total number of pseudopods analyzed. b), Sometimes cells extend two or more “one front” pseudopods with some overlap of their extension period. The counts are the time that cells have two extending fronts and is the sum of the seconds that “Y-split” pseudopods extend with two fronts + the seconds that cells have two or more extending “one-front” pseudopods; the percentage is this sum divided by the total time of pseudopod extension. These raw data are reported here for the first time. Since the data are simple counts, there is no statistics.

Experiments show that pseudopods are regulated by a coupled excitable system of Ras-GTP and branched F-actin (Fig. 1*C*) (5, 8–10). At the place of the future pseudopod a patch of activated Ras/actin increases in size and intensity during about 3 s, and then the pseudopod starts (5). In buffer, the Ras/actin patch grows symmetrically. In a gradient, the Ras/actin patch receives local information from chemoattractant receptors and therefore grows stronger at the up-gradient side: The pseudopod starts with a bias toward the chemoattractant (Fig. 1*D*). The local information can be diverse leading to chemotaxis, electrotaxis, thermotaxis, *et cetera*.

In the model of Alonso et al., the gradient stabilizes one of the Y-split protrusions after the pseudopod started, and therefore, the steepness of the gradient affects the extension time (3). This is not observed in experiments (Table 1). In our model, the gradient influences the start position before the pseudopod emerges; the pseudopod itself is not different in buffer or chemoattractant (Table 1). We conclude that the predominant mechanism for chemotaxis is the local stimulation of the Ras/actin excitable system. In the rare cases that cells extend multiple pseudopods, retraction of the pseudopod not facing the gradient may contribute to orientation in shallow gradients.
